# Cost-effectiveness of collaborative care for the treatment of major depressive disorder in primary care. A systematic review

**DOI:** 10.1186/1472-6963-10-19

**Published:** 2010-01-19

**Authors:** Kirsten M van Steenbergen-Weijenburg, Christina M van der Feltz-Cornelis, Eva K Horn, Harm WJ van Marwijk, Aartjan TF Beekman, Frans FH Rutten, Leona Hakkaart-van Roijen

**Affiliations:** 1 Netherlands Institute for Mental Health and Addiction (Trimbos-Institute), Utrecht, The Netherlands; 2 Institute for Medical Technology Assessment, Erasmus University Medical Centre, Rotterdam, The Netherlands; 3 Institute for Extramural Research, VU University Medical Centre, Amsterdam, The Netherlands; 4 Department of Psychiatry, VU University Medical Centre, Amsterdam, The Netherlands; 5 Onze Lieve Vrouwe Gasthuis, Amsterdam, The Netherlands; 6 EMGO Institute, VU University Medical Centre, Amsterdam, The Netherlands; 7 Department of General Practice, VU University Medical Centre, Amsterdam, The Netherlands

## Abstract

**Background:**

The effectiveness of collaborative care for patients with major depressive disorder in primary care has been established. Assessing its cost-effectiveness is important for deciding on implementation. This review therefore evaluates the cost-effectiveness of collaborative care for major depressive disorder in primary care.

**Methods:**

A systematic search on economic evaluations of collaborative care was conducted in Pubmed and PsychInfo. Quality of the studies was measured with the Cochrane checklist and the CHEC-list for economic evaluations. Cost-effectiveness and costs per depression-free days were reported.

**Results:**

8 studies were found, involving 4868 patients. The quality of the cost effectiveness studies, according to the CHEC-list, could be improved. Generally, the studies did not include all relevant costs and did not perform sensitivity analysis. Only 4 out of 8 studies reported cost per QALY, 6 out of 8 reported costs per depression-free days. The highest costs per QALY reported were $49,500, the highest costs per depression-free day were $24.

**Conclusions:**

Although studies did not fulfil all criteria of the CHEC-list, collaborative care is a promising intervention and it may be cost-effective. However, to conclude on the cost-effectiveness, depression research should follow economic guidelines to improve the quality of the economic evaluations.

## Background

Annually, seven percent of the adults suffer from the consequences of depressive disorder and 16 percent once in their lifetime [1]. In 2020, depression is expected to rank second, after cardiovascular disease, in terms of driving the loss of Disability Adjusted Life Years (DALYs) [2]. Although several effective treatment options for depression are available, their impact on the societal burden or costs of depression remains limited [3]. In depressive disorder, a multidisciplinary approach for treatment may be needed, such as disease management. Disease management programs (DMPs) organise healthcare around a specific disease, i.e. depressive disorder, and provide evidence-based treatment as described in multidisciplinary guidelines [4]. DMPs have been proven effective for the treatment of depressive disorder in US primary care in terms of symptom reduction, quality of life, adherence to medication and attaining remission of depression [5-11]. Collaborative care has important characteristics of a DMP and is used for depression management in the US. It organises care around a patient, using a care-manager to give less-costly, qualitative good and effective care.

In a stepped-care arrangement, the intensity or complexity of care is stepped-up only when proven necessary. Patients are first offered an intervention that, while likely to be effective, is relatively easy to implement and carries relatively low cost or side effects. If the effect turns out to be insufficient, treatment is stepped up to a more complex, costly or taxing (in terms of side effects) level. The aim is to ensure that all eligible patients have access to appropriate care, while reserving the most complex treatments for those that have demonstrated not to benefit for more simple treatment. This strategy can be integrated rather easily within collaborative care, which has proven to be an effective treatment model for the treatment of major depression in primary care. Overall effect sizes range from 0.25 (95% CI 0.18-0.32) in the US [9,12,13] to 0.63 (95% CI 0.18-1.07) in the UK health care system [14]. The longer-term (4 years) effect size was 0.15 (95% CI 0.001-0.31) [15]. Comparing the costs and benefits of collaborative care is necessary when health policy makers request information on the relative efficiency of health care programs. Two 2006 reviews [8,9] addressed presented data on collaborative care, but described DMPs in general and collaborative care as a part of disease management, instead of collaborative care alone. Also, these reviews included studies targeted to healthcare professionals. In the present study, only studies that provided care for patients were included. Besides that, the mentioned reviews provided data of studies that were published until 2005. Several new studies have added economic information, and an update on the collaborative care data therefore is necessary. The aim of this study is to addresses the cost-effectiveness of collaborative care for the treatment of major depressive disorder in primary care.

## Methods

The method of a systematic review is followed [16] and a QUOROM flowchart is used [17]. Cost data were synthesized using descriptive methods.

### Search methods

The search on Pubmed (Medline) and PsychInfo was conducted from October 2007 through June 2008 to include all articles on the subject. Also, the Cochrane Library was checked for reviews or ongoing reviews. An update on the search was performed in October 2009. The search was performed with the following terms: depression, depressive disorder, collaborative care, disease management, stepped care, cost-effectiveness, cost-utility, and cost-benefit analysis and economic evaluation. If a review was found on the subject, the references were checked for eligible studies. No limitations were used.

### Inclusion criteria

The articles had to be a RCT or systematic review of sufficient quality according to the Cochrane criteria [16], and the abstract and full text of the articles had to be available. The cost-effectiveness and, whenever possible, the cost-utility had to be presented. Patients had to be diagnosed with major depressive disorder (MDD), according to the Diagnostic and Statistical Manual of Mental Disorders or International Statistical Classification of Diseases (DSM or ICD) criteria. Articles where co-morbidity of MDD with other diseases was studied were accepted but the focus of the intervention had to be on depressive disorder. Decision-analytic models were not included in this study, as they did not report on the cost-effectiveness of collaborative care.

The intervention had to be based on (stepped) collaborative care for the treatment of major depressive disorder in primary care. Programs were defined as collaborative care if treatment complied with at least three of the following criteria:

1. Within collaborative care the role of care manager is introduced to assist and manage the patient by providing structured and systematic interventions.

2. A network is formed around the patient with at least two of the three following professionals: general practitioner, care manager, and consultant psychiatrist, a definition provided by Katon *et al *[18,19].

3. Process and outcome of treatment is being monitored and in case of insufficient improvement treatment may be changed according to the principles of stepped care [4].

4. Evidence-based treatment is provided [4].

### Data analytic procedures

#### Data abstraction

The abstracts of selected articles that seemed relevant for this review were retrieved and read by two independent reviewers. Based upon the inclusion criteria, they both selected eligible articles. After that, the full text was read and a selection of eligible articles was made. In case of disagreement, a third reviewer decided on the inclusion of the article. When the two independent reviewers both accepted a study, the Cochrane checklist for quality assessment of articles was used [16] to rate the quality of the studies independently and in duplicate. With this checklist, studies can be rated on the presence of randomization and concealment of allocation, the blinding of patients, care providers and outcome assessors, mentioning drop-out and performing an intention-to-treat analysis.

Also, the CHEC-list [20] for economic evaluations was used to assess the quality of the cost-effectiveness measurements. In this CHEC-list, 19 points can be scored when aspects are properly addressed in the article, for instance a good description of the study population, the intervention and competing alternatives, the research question and the perspective from which the costs and effects are measured and whether relevant costs are measured, relevant outcomes are defined, an incremental analysis is performed, the generalizability is discussed and a sensitivity analysis is performed. A well performed economic evaluation adequately addresses all 19 items in the article.

#### Effects

The effects were measured with validated depression scales e.g. the Hamilton Rating Scale for Depression (HRSD), the Hopkins Symptom checklist (HSCL) or the Montgomery Åsberg Depression Rating Scale (MADRS). More general effect measurements included Quality Adjusted Life Years (QALYs) and days absence from work.

#### Costs

Preferably, costs are measured from a societal perspective, including all relevant costs for society. The societal costs can be divided into direct medical costs e.g. outpatient costs, medication and hospital days, direct non-medical costs e.g. travelling costs, and indirect non-medical costs e.g. the costs caused due absence of work and total costs. The costs can be measured from tariffs (billed charges), or actual costs. Tariffs are the maximum costs that for example a specialist may charge for a consult, actual costs are the costs a specialist makes for each consult including personnel and overhead. All costs were measured in US dollars, or converted to US dollars if necessary, using the currency rate at the time the study was performed. There was no adjustment for different financial years.

#### Cost-effectiveness

Costs per QALY is the preferred unit for measuring cost-effectiveness. This is an international used measurement with accepted cut-off points. A UK approach, by Devlin & Parkin [21], states that a new intervention is cost-effective when it costs a maximum of £20,000 - £30,000 per Quality Adjusted Life Year (QALY); approximately US $34,000 - $51,000. In depression-management it is usual to measure the costs per depression-free day (DFD) or the costs per successfully treated patient. However, there is no consensus about the societal accepted value of a DFD.

#### Analyses

First, the studies were assessed according to the CHEC-list for economic evaluations [20]. Subsequently, effect measurements, cost components, perspectives and cost-effectiveness ratios were derived from the studies, reported in values of the same year of the trials. Findings of cost-effectiveness were presented in a permutation matrix, adopted from Nixon *et al *[22]. In this permutation matrix, the costs and effects of the intervention group are compared with the costs and effects of the control group. The results are reported in a descriptive manor, only showing the most striking results, as there were too many to report all and too differently measured to aggregate them.

## Results

### Study selection

**Figure 1:**
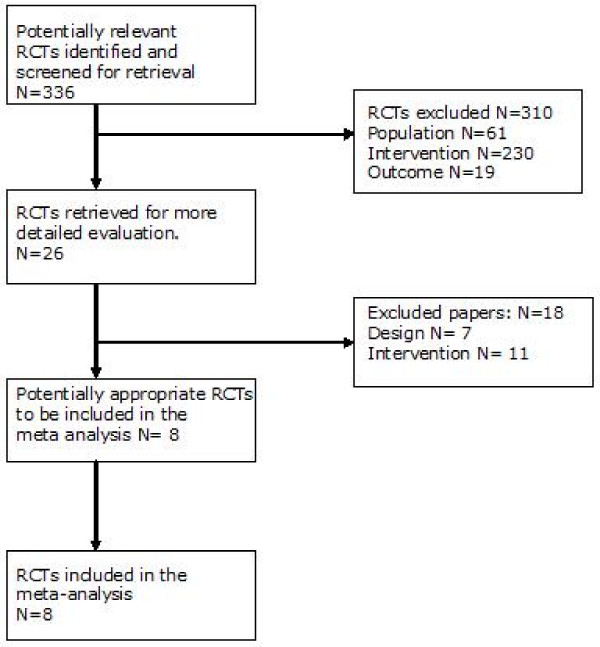
**Flowchart (Moher *et al ***[17]**)**.

In figure 1, a flowchart of the study selection is presented [17].

From the initial search (336 abstracts), 26 studies were selected based on the abstracts. Based on full text examination, 18 studies were excluded; 4 were study-protocols, 13 did not meet the inclusion criteria and 1 was a systematic review that reviewed articles in our selection. All studies fulfilled the preset criteria for treatment according to collaborative care. All eight remaining studies were deemed of sufficient quality, as shown in table 1[23-30] There was no disagreement between the two assessors. Using the 19-item CHEC-list [20] the included studies were assessed on quality of performed economic evaluations. A maximum of 10 points out of 19 was scored, as shown in table 2.

**Table 1:** Quality assessment of the RCTs by using the Cochrane Quality checklist[16].

Article	Quality grade (out of 7) By reviewer:		Article included/excluded
	KW	CFC	
Von Korff *et al*[23]	4	4	Included

Simon *et al*[24]	5	5	Included

Simon *et al*[25]	6	6	Included

Schoembaum *et al*[26]	4	4	Included

Liu *et al*[27]	4	4	Included

Araya *et al*[28]	4	4	Included

Katon *et al*[29]	4	4	Included

Simon *et al*[30]	5	6	included

Cochrane Quality Checklist: randomized allocation of participants, concealed treatment allocation, blinded patients, blinded caregivers, blinded outcome assessors, drop-out rate acceptable, intention-to-treat analysis performed.

**Table 2:** Quality assessment of the economic evaluations by using the 19-item CHEC-list[20].

Study	Score on the 19-point CHEC-list for economic evaluations	
	KW	LH
Von Korff *et al*[23]	6	5

Simon *et al*[24]	7	6

Simon *et al*[25]	9	6

Schoenbaum *et al*[26]	9	7

Liu *et al*[27]	7	6

Araya *et al*[28]	9	7

Katon *et al*[29]	8	5

Simon *et al*[30]	10	9

CHEC-list: study population, competing alternatives, research question, perspective, relevant costs, relevant outcomes, incremental analysis, generalizability, sensitivity analysis.

In total, eight studies were included, all providing cost-effectiveness analyses of a collaborative care intervention for the treatment of major depressive disorder. While this was not a restriction, all studies were performed in primary care [23-30]. Except for two [23,24], all studies measured the treatment effect using average depression-free days. The costs were measured and presented in direct medical costs and direct non-medical costs. Four studies analysed both cost-effectiveness and cost-utility [24-26,29], the other studies performed a cost-effectiveness analysis not considering QALYs [23,27,28,30].

### Study outcomes

The main findings of the included studies are summarized in table 3.

**Table 3:** Cost-effectiveness of primary care interventions based on collaborative care.

First author	Population	Accepted as CC because:	Cost measurement	Effectiveness measurement	Results
Von Korff *et al*, 1998[23] Prices based on the year: unknown	Primary care patients (US), newly diagnosed major depression (DSM-III-R), N = 153 Mean age: I = 43.1, C = 44.8	Collaborative management, psychologist and physician, problem-solving treatment	Health care perspective Intervention costs, primary care costs Follow-up: 7 months	SCL-90 score, successfully treated case of depression	Incremental cost-effectiveness: $3741 per successfully treated case of major depression. Reduction in SCL-score (70.4% vs. 42.3%) Conclusion: strong indication for cost-effectiveness

Simon *et al*, 2001a[24] Prices based on the year: 1996	Patients not responding to 8 weeks usual care, US primary care, major depression according to DSM-IV, N = 228 Mean age: I = 45.6, C = 45.4	Stepped care, patient education, advice to patient and physician by psychiatrist, ongoing management	Social perspective Intervention costs, non-depression primary care costs, total out-patient treatment costs, total health service costs Follow-up: 6 months	SCL-90 score, HRQoL	Incremental intervention related direct medical costs: $21 per DFD (95% CI $8 to $126) over 6 months. Total healthcare costs $35 per DFD (95% CI -$52 to $388) over 6 months. Patient costs: travelling expenses I = $1636 (95% CI 1356-1916) C = 1337 (95% CI 1174-1499) I = 50% reduction SCL score, 95% CI 1.02 - 2.03. Conclusion: strong indication for cost-effectiveness

Simon *et al*, 2001b[25] Prices based on the year: unknown	High utilisers of medical care, high probability of undiagnosed major depression (DSM-IV), US primary care, N = 407. Mean age: I = 47.2, C = 46.7	Depression management coordinated by primary care mental health worker, psychiatrist support, physician and patient education, using guidelines	Health care perspective Intervention costs, in- and outpatient depression treatment costs Follow-up: 12 months	HRSD improvement, HRQoL,	Intervention related direct medical costs $21 (95% CI $11 to $38) per DFD. HRSD: 53.2% (I) vs. 32.8% (C) shows 50% improvement after 12 months Conclusion: strong indication for cost-effectiveness

Schoenbaum *et al*, 2001[26] Prices based on the year: 1998	US primary care, major depression according to DSM-IV, N = 1356 Mean age: I = 44.5, C = 42.2	Nurse specialists follow-up patients, conjunction between nurse - primary care physician and specialist	Health care perspective Depression primary care costs, intervention costs, costs per QALY Follow-up: 24 months	HRQoL (QALYs calculated from SF-12 and Depression burden days), CES-D	Intervention related direct medical costs by SF-12 method $21 478 per QALY (confidence interval not given). By DFD method, 95% CI $9478 to $18 953 CES-D reduction: 50% for intervention. Conclusion: strong indication for cost-effectiveness

Liu *et al*, 2003[27] Prices based on the year: 2000	Veteran population, Male = 95%, Major depression according to DSM-IV, N = 354 Mean age: I = 57.8, C = 56.6	Patient education, progress evaluation, team meetings, stepped care	Health care perspective Total (outpatient) costs Follow-up: 9 months	SCL-90 score	DFD increment for I = 14.6 Intervention related direct medical costs: I = $615 higher, $3754 (CI 3329-4179) vs. $3139 (CI 2759 -3519). Total intervention costs: I = $1259 higher; $7946 (CI 5582-10310) vs. $6789 (CI 4720-8858). I = SCL sign. better at 3 months (P < .25) Conclusion: strong indication for cost-effectiveness

Araya *et al*, 2006[28] Prices based on the year: 2004	Females 18-70 years, major depression according to DSM-IV, N = 240 Mean age: I = 44.1 (SD 12.1) and C = 42.0 (SD 13.7)	Monitoring clinical progress, manager coordinates with physicians, stepped care	Health care perspective Costs per patient, cost-effectiveness ratio Follow-up: 6 months	HRSD score	DFD: I = 50 more. Incremental cost-effectiveness ratio = 0.75. Intervention related direct medical costs: I = $87.8 per patient (CI 78.9 - 103.4), C = $51.5 per patient (CI 43.0 - 60.5) Conclusion: cost-effective

Katon *et al*, 2005[29] Prices based on the year: unknown	Diabetes, major depression according to DSM-IV, N = 1801 Mean age: I = 71, C = 71.4	Based on IMPACT protocol, stepped care, depression care manager consults with professionals, problem-solving treatment	Health care perspective Outpatient mental health costs, costs per QALY Follow-up: 24 months	HSCL-20 improvement, QALYs	Increment in DFD 1^st ^year: 52.6 (CI 42.2 - 63.0). Increment in DFD 2^nd ^year: 54.3 (CI 42.2 - 66.2). Intervention related direct medical costs 2^nd ^year follow-up = $921 higher than CAU. Costs per QALY: $2519 (95% CI -$4517 to $9554)) to $5037 (95% CI -$9034 to $19 108). Conclusion: strong indication for cost-effectiveness

Simon *et al*, 2007[30] Prices based on the year: unknown	Diabetes and major depression according to DSM-IV, N = 329 Mean age: I = 58, C = 57	Stepped care, depression nurse coordinates contacts with professionals and patient, treatment based on the IMPACT-model	Health care perspective Outpatient costs Follow-up: 24 months	SCL-90 score	Increment in DFD: I = 20 in 1^st ^year, 33 in 2^nd ^year. Intervention related direct medical costs costs: I = $1400 lower in 2^nd ^year, mean I = $600 lower in 2^nd ^year. Health services in 2 years: I = $21148 (SD 27548) vs. C = $22258 (SD 35607) Conclusion: strong indication for cost-effectiveness

C = control, CC = collaborative care; CE = cost-effectiveness; CES-D = Centre for Epidemiologic Studies-Depression scale; DFD = depression free days; DSM = diagnostic and statistical Manual of mental disorders; E = effectiveness; HRQoL = health related quality of life; HRSD = Hamilton Rating Scale for Depression; HSCL-20 = Hopkins symptom checklist -20; I = intervention; MADRS = Montgomery Åsberg depression rating scale; PRIME-MD = primary care evaluation of mental disorders; QALY = quality adjusted life year; SCL-90 = Symptom checklist - 90; SF-12 = Short Form

### Demographic variables

The population as described in the included studies, has the following characteristics. One study[29] included patients aged 60 or more; other studies covered a general adult population. The mean age reported varies from 40 to 71.2 years old.

The total amount of participants (all eight studies combined) was 4868 and the lowest number reported in one study was 145. One included a veteran population, mainly consisting of males (95%) [27] and one study investigated the collaborative care intervention for patients with major depressive disorder and diabetes mellitus[30]. Seven studies were performed in the United States [23-27,29,30] and one was performed in Chile [28]. All studies fulfilled the criteria for collaborative care and all studies compared collaborative care to care as usual in primary care.

### Effects

#### Depression-free days

Most of the studies reported depression-free days as the primary outcome measure [24,25,27-30]. These were calculated by using the Hamilton Rating Scale for Depression (HRSD) where a score equal or less to 7 is a depression free day [24,28]. Others used the Symptom Checklist (SCL-90 or HSCL-20) where a score of .5 or less is considered depression free [25,27,29,30]. Both 'translations' into depression free days are reported by Lave *et al *[31]. The follow-up of the included studies varied from 6 months to 24 months.

The study by Liu *et al *[27] reported an increment in depression-free days of 14.6 in a nine-month follow-up, while the other studies reported 20 to 60 more depression-free days in the first year of the follow-up, compared to the control group [24,28-30]. In the second year of the follow-up, the intervention group reported 33 to 56 more depression-free days compared to the control group [28-30].

Two studies reported reductions in SCL-score [23,27] but only one [23] reported numbers: a 50 percent reduction for the intervention group. For the care as usual group, a 40 percent reduction was reported [23]. Liu *et al *[27] only reported that there was a significant reduction in SCL-score at three months (P < .025), at 9 months there was no significant effect.

#### QALYs

Four studies reported QALYs next to depression-free days, over a average period of 6 months. In three studies [24,25,29] these were calculated based on the change from the HRSD-score or SCL-score. The improvement from depression to remission was associated with an improvement of 0.2-0.4 in the measure of QALY, as defined in previously performed cost-utility studies [32-34]. The number of QALYs calculated with this method varied from 0.05 [23] to 0.117 [29]. Schoenbaum *et al *[26] calculated QALYs in two ways: from the Short Form-36 (SF-36) and the depression-burden (DB) days. The amount of QALYs were derived from a sample of primary care patients with symptoms of depression, using a standard gamble approach [26]. The SF-36 method calculated an increase in QALYs of 0.0226 (P = .006) due to the intervention and the DB method calculated an increase of 0.0258 to 0.0515 QALY, according to Schoenbaum *et al *[26].

All studies claimed that collaborative care for the treatment of depressive disorder is more effective than care as usual in terms of depression-free days and QALYs.

### Costs

#### Direct medical costs

Most studies were conducted from a health care perspective, only two included production losses and travelling expenses. The studies were performed in primary care and reported health service use in primary care, outpatient specialty mental healthcare and admissions, as reported in table 4. The costs were measured by multiplying the measured healthcare usage and the charges for these healthcare services. Across the various studies, this is done by self-reported healthcare service usage [26,28], by records of patient contacts [23,27,29], computerized health plan data [24,25,30].

**Table 4:** Incremental costs and depression-free days.

Study	Incremental DFDs for CC vs. CAU	Incremental costs for CC vs. CAU in US$	Costs measurement
Von Korff *et al*[23]	None reported	3741	Intervention costs, primary care costs (average direct and overhead costs to the health maintenance organization)

Simon *et al*[24]	47.4	1640	Total health services costs (anti-depressants, outpatient specialty services, inpatient services, intervention costs)

Simon *et al*[25]	16.8	296	Health services costs (outpatient costs, inpatient costs mental health specialty costs, medications, visits CC psychiatrist)

Schoenbaum *et al*[26]	None reported	485	Intervention costs, healthcare costs (emergency department visits, medical and mental health visits, and psychotropic medications used)

Liu *et al*[27]	14.6	1157	Total health services costs (antidepressants, primary care visits, mental health specialty care, intervention program costs)

Araya *et al*[28]	50	36.4	Total health services costs (Medical consultation costs, medication use, use of health services, intervention costs)

Katon *et al*[29]	107 (2 years follow-up)	921 (two years follow-up)	Total health services costs (anti-depressants, intervention costs, outpatient specialty mental health care costs)

Simon *et al*[30]	20	26	Total outpatient costs (outpatient depression treatment costs and outpatient non-depression treatment costs)

DFD = depression-free day; CC = collaborative care; CAU = care as usual.

Four studies mentioned intervention related costs. In the study by Katon *et al *[29] the increment in intervention related direct medical costs was $921 per person for a two year follow-up, compared with CAU.

Simon *et al *[30] reported intervention related direct medical costs over 2 years of $21,148 (SD $27,548) for the intervention group vs. $22,258 (SD $35,607) for the control group, a saving of $1,110.

In the study by Liu *et al *[27] the increment in intervention related direct medical costs was $519 for the intervention group, compared with CAU.

The study by Simon *et al *[24] reported an increment in intervention related direct medical costs of $675. For outpatient costs (all contacts with medical or ancillary providers and medications) and inpatient health services costs (hospitalization, inpatient services and procedures, medications) together, the increment was $1,974.

#### Direct non-medical costs

In the study by Simon *et al and *Schoenbaum *et al *[24,26], the follow-up assessments also included the time costs (including travel and waiting time), for obtaining healthcare. The time for outpatient medical and mental health visits was, respectively, 30 and 45 minutes [26].

The travelling expenses varied from $1,636 (95% CI $1,356-$1,916) for the intervention group to $1,337 (95% CI $1,174-$1,499) for the CAU group [24].

#### Indirect non-medical costs

One study reported production losses, measuring the days worked in 6 months and the days missed from work due to illness [26]. There was a significant intervention effect on days worked overall, but the amount of sick days among workers did not statistically differ between the intervention and usual care patients. None of the included studies had quantified the production losses into costs.

### Cost-effectiveness

All studies found that collaborative care is effective but in most cases also more expensive than CAU [23-27,29,30]. In one study, the total outpatient costs in the first year of the follow-up were higher compared to care as usual. However, in the second year of follow-up costs of the intervention were lower [30]. Another study [28], conducted in Chile, collaborative care was significantly more effective and marginally more expensive than usual care after 6 months of follow-up.

Six studies reported the intervention related direct medical costs per depression-free day [24,25,27-30]. The increment in costs for the intervention group compared with the control group varied from $20 to $24 per depression free day [24,27]. The incremental cost-effectiveness ratio (the healthcare costs per depression-free day) was reported by Katon *et al *[29] at 2.76. One study included other direct medical costs (medications, total medical consultations, healthcare usage) [28] and calculated the incremental cost-effectiveness ratio at 0.75 in Chilean Pesos.

Von Korff *et al *[23] reported the costs per successfully treated patient, in this case a 50% or greater reduction in SCL-score at 4-month follow-up. The average costs per successfully treated patient were $1,797 for the intervention group vs. $1,941 for the control group.

### Cost- utility

**Figure 2:**
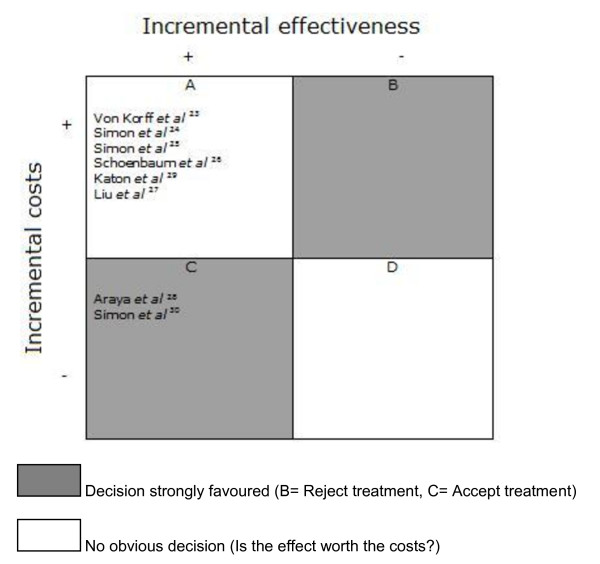
**Permutation matrix for the cost-effectiveness of collaborative care vs. care as usual**. (Adopted from Nixon *et al *[22]).

Four studies presented cost-utility ratio's in which incremental costs per QALY were estimated [24,26,28,29]. The direct medical costs per QALY are divided into intervention costs and healthcare costs. The intervention costs varied from $2519 (95% CI -4,517 - $9,554) to $5037 (95% CI -$9,034 to $19,108) [29]. The direct- and indirect costs per QALY together were $21,478 [26]. Simon *et al *[24] measured the costs per QALY at $49,500. The findings are summarized in figure 2, where the costs and effects for the intervention group (in comparison with the control group) per study are compared.

## Discussion

The included studies scored a maximum of 10 points out of 19 on the CHEC-list, indicating that the quality of the studies can be improved. Studies on the cost-effectiveness of collaborative care indicate that this type of intervention for the treatment of MDD in primary care can be cost-effective, relative to usual care, depending on willingness to pay. The collaborative care patients have 60 more depression-free days than care as usual patients, and in the second year of the follow-up this is 56 depression-free days. For the collaborative care interventions on the treatment of depressive disorder, the reported incremental costs per QALY were $21,478 to $49,500 for all the costs of healthcare services together.

But to determine the cost-effectiveness of collaborative care, it is necessary to compare the costs of the intervention with the benefits. What can be helpful is to look at previously performed studies of which the intervention has been implemented. For example the study of Devlin and Parkin [21], where they compare studies that have been implemented by the NICE institute in Great Britain. They concluded that when the costs per QALY did not exceed the maximum of $51,000, the study was implemented [21].

Unützer *et al *[15] have reported the long term costs of collaborative care treatment. In a 4-year follow-up of 551 patients the direct medical costs (here the costs of the intervention, outpatient costs, pharmacy costs and inpatient costs together) were measured. The intervention group accounted for $29,422 (95% CI $26.479 - $32,365) versus $32,785 (95% CI $27.648 - $37.921) for the control group; a difference of $3,363 in favour of the intervention group.

### Limitations of the study

The quality of the economic evaluations was assessed with the CHEC-list [20]. Items on the CHEC-list that scored low were among others the performance of sensitivity analysis and the usage of QALYs. Costs of sick leave were only measured in one study, while studies showed that productivity costs are responsible for a majority of the costs [35,36]. Also, studies were only performed for a maximum of 6 months, which leaves the long-term consequences unknown. Unfortunately only 4 out of 8 studies considered the costs per QALY. Future studies on the cost-effectiveness of collaborative care should be performed according to an economic guideline to have good quality studies [37]. The $51,000 cut-off point for costs per QALY is not optimal as this is a UK recommendation while most studies were performed in the US. Every country that wants to implement collaborative care has to define itself which cut-off point they use, based on the willingness to pay of their citizens.

Furthermore, younger adults were underrepresented in the review as most studies described an older population (with a minimum age of 50 years old). Because of the population, indirect non-medical costs were not measured as they were relatively irrelevant in the medically ill, elderly or war veteran groups. However, the average clinical population is typically in their mid 40's, so it affects the generalizability of these study results to the average population. The assessment of the direct non-medical costs (traveling expenses) and indirect non-medical costs (production losses) needs to be improved. Also, the follow-up of the studies varied and this may cause a difference in costs. All this may have a significant impact on the total costs [33].

Thirdly, most studies are conducted in the US, in a MCO or at Kaiser Permanente departments in different parts of the US. This may affect the generalizabiliy of the studies to other countries or healthcare systems. Several European countries (the Netherlands and Hungary for instance) have reformed their health care system recently, and it can be very interesting to see if collaborative care is cost-effective in these healthcare systems. Also, all studies were performed in primary care, but the question is how it will translate to for instance the general hospital.

Finally, the definition of collaborative care is not uniform. Not every study labelled their intervention collaborative care, but they did meet the inclusion criteria of collaborative care. It merits recommendation to make one definition to make future research comparable.

## Conclusions

Collaborative care seems a promising option to deliver the right treatment for patients with major depressive disorder. However, the economic information is insufficient for policy decisions. The number of studies on cost-effectiveness of collaborative care is still limited and the quality of these economic evaluations should be improved. For example, only a limited number of studies presented the cost-effectiveness in terms of costs per QALY and generally costs-effectiveness was reported as the costs per DFD. Information on the (cost-) effectiveness of CC in other settings than primary care is needed before CC can be broadly implemented.

## Competing interests

The authors declare that they have no competing interests.

## Authors' contributions

KvS analysed data and wrote the article, CFC supervised and reviewed the article, EH reviewed the article and collected data, HvM reviewed the article, AB supervised and reviewed the article, FR supervised and reviewed the article, LHR supervised data analysis and reviewed the article. All authors read and approved the final manuscript.

## Pre-publication history

The pre-publication history for this paper can be accessed here:

http://www.biomedcentral.com/1472-6963/10/19/prepub
